# Transcriptomic Analysis of Muscle Satellite Cell Regulation on Intramuscular Preadipocyte Differentiation in Tan Sheep

**DOI:** 10.3390/ijms26073414

**Published:** 2025-04-05

**Authors:** Xiaochun Xu, Cong Zhan, Jiaqi Qiao, Yuxuan Yang, Changyuan Li, Pan Li, Sen Ma

**Affiliations:** 1 Collaborative Innovation Center for Food Production and Safety, School of Biological Science & Engineering, North Minzu University, Yinchuan 750021, China; zhancong0301@163.com (C.Z.); 15504729932@163.com (J.Q.); 18295400901@163.com (Y.Y.); 15338619561@163.com (C.L.); leepanan@163.com (P.L.); 2 College of Animal Science and Technology, Henan Agricultural University, Zhengzhou 450002, China

**Keywords:** Tan sheep, muscle satellite cells, intramuscular preadipocyte, ligand–receptor pair, cell communication

## Abstract

Intramuscular fat (IMF) content is a key factor influencing meat properties including tenderness, flavor, and marbling. However, the complex molecular mechanisms regulating IMF deposition, especially the interactions between intramuscular preadipocytes (IMAdCs) and skeletal muscle satellite cells (SMSCs), remain unclear. In this study, a direct co-culture system of sheep IMAdCs and SMSCs was used to elucidate their intercellular interactions. RNA sequencing and bioinformatics analyses were performed under monoculture and co-culture conditions for later stages of differentiation. The obtained results showed that SMSCs significantly inhibited the adipogenic capacity of IMAdCs. This was reflected in the co-culture markedly altered gene expression and observations of lipid droplets in our studies, i.e., the *PPARG*, *ACOX2*, *PIK3R1*, *FABP5*, *FYN*, *ALDOC*, *PFKM*, *PFKL*, *HADH*, and *HADHB* genes were down-regulated in the co-cultured IMAdCs in association with the inhibition of fat deposition, whereas *ACSL3*, *ACSL4*, *ATF3*, *EGR1,* and *IGF1R* within the genes upregulated in co-culture IMAdCs were associated with the promotion of lipid metabolism. In addition, GO, KEGG, and ligand–receptor pairing analyses further elucidated the molecular mechanisms of intercellular communication. These findings emphasize the regulatory role of SMSCs on intramuscular preadipocyte differentiation and lipid metabolism, providing a theoretical framework for targeted molecular strategies to improve sheep meat quality.

## 1. Introduction

The intramuscular fat (IMF) level has a positive impact on the eating quality of meat, as an increased content of IMF improves meat quality, particularly tenderness, flavor, and overall consumer preference [1]. IMF refers to the fat content within muscle tissue, and its variation among animals is influenced by genetics, development, nutrition, sex, age, and management practices. Additionally, early developmental processes, including adipocyte proliferation and differentiation, as well as the structure of surrounding connective tissue, contribute to individual differences in IMF levels [2]. A factor influencing IMF variation is the differential expression of genes involved in fatty acid metabolism during early development, which has been linked to IMF content at slaughter [3]. Variations in IMF content are primarily influenced by the number and size of intramuscular adipocytes, while the rate of IMF accumulation is closely associated with muscle growth dynamics. For instance, animals with greater muscle mass and higher glycolytic activity typically exhibit lower IMF accumulation, indicating a dynamic interaction between muscle cells and adipocytes during growth [4].

Intercellular communication represents a fundamental biological process, with emerging evidence suggesting potential crosstalk mechanisms between intramuscular preadipocytes (IMAdCs) and skeletal muscle satellite cells (SMSCs). Previous studies have demonstrated species-specific regulatory effects. For instance, co-culture of bovine SMSCs with preadipocytes significantly upregulated adipogenic marker genes, such as C/EBPβ and PPARγ, in myocytes [5]. In contrast, experiments on mice revealed that satellite cell-derived myofibers inhibit the adipogenic differentiation of mesenchymal progenitors [6], a phenomenon further corroborated in porcine SMSC–preadipocyte co-culture systems [7]. The shared mesenchymal stem cell origin and anatomical proximity between SMSCs and IMAdCs provide a strong theoretical foundation for potential cellular crosstalk. Despite this, systematic investigations into the interaction mechanisms between IMAdCs and SMSCs in livestock remain largely unreported, highlighting a critical gap in current research. The co-culture strategy provides a powerful opportunity for deeply investigating interaction mechanisms between myocytes and adipocytes [8]. Notably, preadipocyte and myocyte precursors share a common mesodermal origin and are regulated through autocrine, paracrine, and endocrine signaling pathways [9]. Co-culturing these progenitor cells offers a particularly effective approach for elucidating biological regulatory processes during development. Previous studies have demonstrated that intramuscular preadipocytes co-cultured with satellite cells exhibit reduced lipid droplet accumulation and decreased intracellular lipid deposition compared to monocultured counterparts, alongside significant downregulation of pro-adipogenic regulatory factors [10]. Mechanistically, myostatin has been shown to suppress adipogenic differentiation in 3T3-L1 cells via miR-124-3p-mediated GR inhibition [11]. However, due to species-specific variations, it remains unclear whether similar molecular pathways govern SMSC-mediated regulation of IMAdC differentiation in other animals. To address this gap, this study establishes a SMSC-IMAdC co-culture system integrated with transcriptome sequencing to systematically explore regulatory effects and molecular mechanisms underlying SMSC-mediated modulation of IMAdC biology in other animals.

The Tan sheep is a famous native sheep breed in China, characterized by a high fat accumulation capacity, superior meat quality, and uniform lipid distribution [12]. Due to its excellent eating quality and pleasant aroma, Tan sheep is considered an ideal material for evaluating and studying mutton quality [13]. Lipids in meat possess unique bioactive properties that influence its quality and acceptability, making them indispensable nutrients for maintaining health. Additionally, as structural components of cell membranes, products or intermediates in signaling pathways, and agents for energy production and storage, lipids play a critical role in various biological systems [14].

This study compared intramuscular preadipocyte cultures from Tan sheep with those co-cultured with skeletal muscle satellite cells at the late differentiation stage. Transcriptomic sequencing was used to screen genes and signaling pathways regulating differentiation and adipocyte deposition. The aim of this study was to investigate the effect of the co-culture system on the differentiation of intramuscular preadipocytes, thereby providing a theoretical foundation for understanding the molecular mechanisms regulating intramuscular fat deposition in Tan sheep.

## 2. Results

### 2.1. Establishment of a Co-Culture System with Intramuscular Preadipocytes and Skeletal Muscle Satellite Cells from Tan Sheep

**Figure 1:**
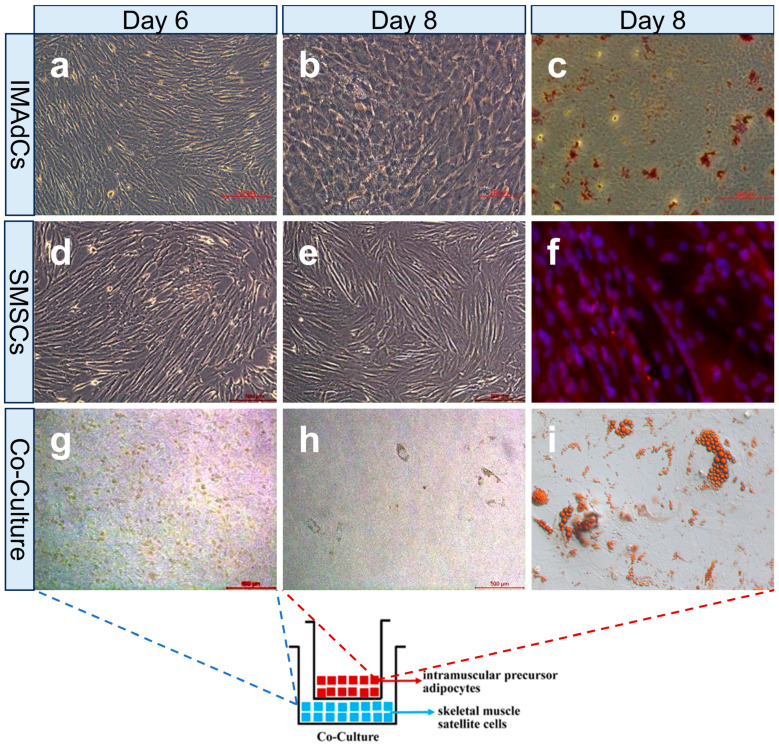
Cell isolation and establishment of the co-cultured system. (**a**) Intramuscular preadipocytes on the 6th day of purification. (**b**) Intramuscular preadipocytes on the 8th day of differentiation. (**c**) Oil Red O staining of intramuscular preadipocytes on the 8th day of differentiation. (**d**) Skeletal muscle satellite cells on day 6 of purification. (**e**) Induced differentiation of skeletal muscle satellite cell on the 8th day. (**f**) Immunofluorescence staining of MyHC and the nucleus on the 8th day of induced differentiation of skeletal muscle satellite cells. (**g**) Sixth-day skeletal muscle satellite cells in the combination culture system. (**h**) Co-cultured intramuscular preadipocytes on the 8th day of induced differentiation. (**i**) Oil Red O staining of co-cultured intramuscular preadipocytes on the 8th day of adipogenic differentiation.

The purified intramuscular preadipocytes developed rapidly and had a long spindle shape after 6 days of incubation (Figure 1a). Lipid droplets were clearly visible after induced differentiation of Tan sheep preadipocytes using adipogenic media for 8 days (Figure 1b). The induced differentiated cells were then identified by Oil Red O staining, with lipid droplets prominently colored red. This showed that the intramuscular preadipocytes were successfully differentiated into mature adipocytes (Figure 1c). Six days later, skeletal muscle satellite cells were isolated from the skeleton, with the cells arranged in a parallel-oriented and tightly packed arrangement (Figure 1d), merging into muscle-generated myotubes. The cell fusion from 8 days later is shown in Figure 1e, and the results reveal that after differentiation on the 8th day, the presence of positive nuclei and the formation of myotubes are clearly observed (Figure 1f).

On the 6th day of co-culture, skeletal muscle satellite cells were evenly distributed, with no obvious lipid droplet accumulation or myotube formation observed (Figure 1g). Lipid droplets were evident after 8 days of induction (Figure 1h), as shown by red adipocytes stained with Oil Red O.

### 2.2. Quantitative Analysis of the Sequencing Data of Tan Sheep and Identification of Differentially Expressed Genes (DEGs)

**Figure 2:**
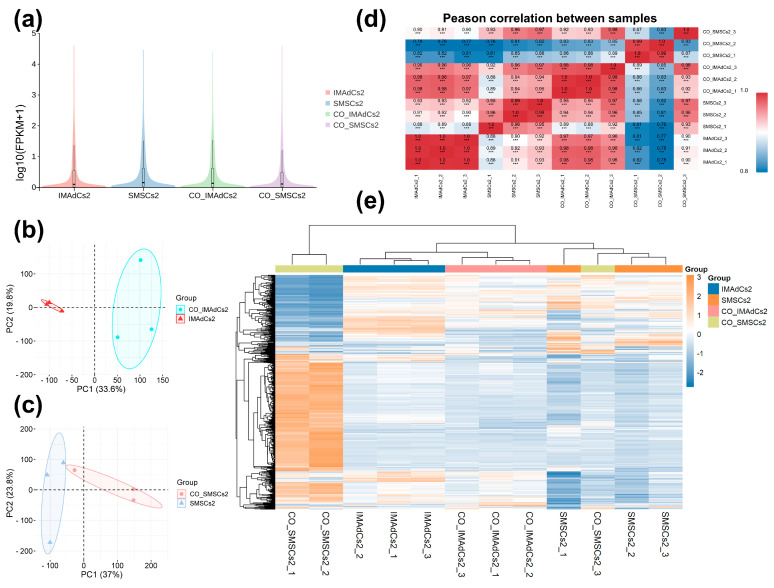
Transcriptome analysis of gene expression patterns in IMAdCs and SMSCs of Tan sheep. (**a**) Violin diagram: Violin plots illustrate the distribution of gene expression levels across sample groups (IMAdCs2, SMSCs2, CO−IMAdCs2, CO−SMSCs2) with log_10_(FPKM+1) transformation. The violin width reflects kernel density estimation, while horizontal lines denote median (solid) and interquartile range (dashed). (**b**,**c**) Principal component analysis (PCA): demonstrates global transcriptional disparities among groups, where scatter points represent individual samples (circles: co−cultured groups; triangles: monocultured groups) with 95% confidence ellipses. (**d**) Heat map of inter-sample correlation: Heatmap visualization of Pearson correlation coefficients between samples employs a blue−to−red gradient to indicate correlation strength. *** *p* < 0.001. (**e**) Heatmap of gene expression between samples: The clustering heatmap of gene expression shows the patterns of genes across sample groups.

In our study, we analyzed the RNA-seq data of intramuscular adipocytes and skeletal muscle satellite cells from Tan sheep samples. After filtering the raw sequencing data, we assessed key quality metrics, including error rate, GC content, and percentages of Q20 and Q30 (Table S1). The violin plot of FPKM values (fragments per kilobase of transcript per million mapped reads) across all samples displays a relatively uniform distribution of gene expression, thereby corroborating the high quality of the sequencing data (Figure 2a). Principal component analysis (PCA) of the whole-transcriptome data, alongside inter-sample correlation analysis and hierarchical clustering of differentially expressed genes (DEGs), was utilized to evaluate the consistency and variability among the twelve experimental samples. PCA demonstrated distinct clustering patterns between co-cultured and monocultured intramuscular preadipocytes (IMAdCs) and skeletal muscle satellite cells (SMSCs) (Figure 2b,c). Pearson correlation coefficient analysis revealed strong intra-group correlations (approaching 1.0) with substantially reduced inter-group correlation values, indicating significant between-group differentiation (Figure 2d). These findings were corroborated by hierarchical clustering results (Figure 2e), collectively validating excellent intra-group reproducibility and marked inter-group heterogeneity across experimental conditions.

**Figure 3:**
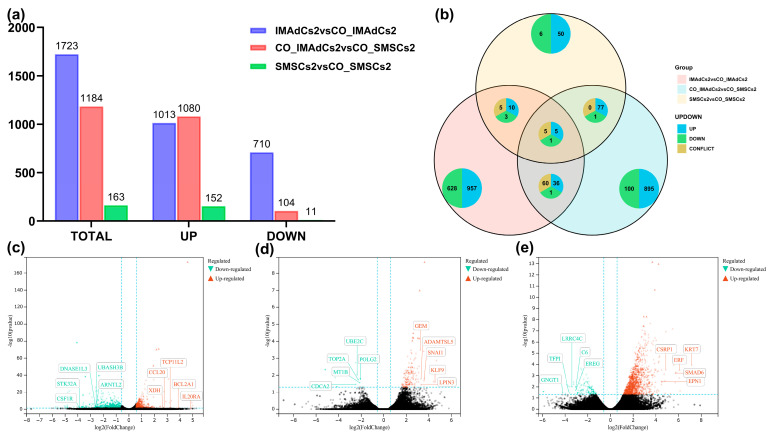
Differential gene expression analysis in Tan sheep IMAdCs and SMSCs under individual and co-culture conditions. (**a**) The bar graph illustrates the number of differentially expressed genes (DEGs) identified in the IMAdCs2 vs. CO_IMAdCs2, CO_IMAdCs2 vs. CO_SMSCs2, and SMSCs2 vs. CO_SMSCs2 comparisons, including upregulated (UP), downregulated (DOWN), and total (TOTAL) gene counts. (**b**) The Venn diagram and pie charts display the overlap and specificity of differentially expressed genes (DEGs) among the three groups. DEGs are categorized as upregulated (UP), downregulated (DOWN), and conflicting (CONFLICT), with colors distinguishing each category. (**c**) The volcano plot illustrates the distribution of differentially expressed genes between the IMAdCs2 and CO_IMAdCs2 groups. The *x*-axis represents the log2 fold change, while the *y*-axis corresponds to the significance (−log10P value). Red triangles indicate upregulated genes, green triangles denote downregulated genes, and significantly altered genes are highlighted. (**d**) The volcano plot displays differentially expressed genes between the SMSCs2 and CO_SMSCs2 groups. (**e**) The volcano plot shows the distribution of differentially expressed genes between the CO_IMAdCs2 and CO_SMSCs2 groups.

To assess the effect of co-culture on gene expression, differential expression analysis of genes (DEGs) between the three sample groups was conducted using DESeq2(Version 1.10.1) [15]. In the comparison between the IMAdCs2 and CO_IMAdCs2 groups, 1723 DEGs were identified, including 1013 upregulated genes and 710 downregulated genes. In the comparison between the CO_IMAdCs2 and CO_SMSCs2 groups, 1184 DEGs were found, consisting of 1080 upregulated genes and 104 downregulated genes. In the comparison between the SMSCs2 and CO_SMSCs2 groups, only 163 DEGs were identified, with 152 upregulated genes and 11 downregulated genes. These results suggested that under co-culture conditions, the transcriptome of IMAdCs2 underwent significant changes, while SMSCs2 showed relatively fewer changes (Figure 3a). Consequently, the focus of subsequent research will be on the effects of co-culture on IMAdCs2. Venn and pie charts illustrate the overlap and specificity of DEGs across the three comparison groups. The pie chart further shows the proportions of upregulated and downregulated genes, with upregulated genes predominating (Figure 3b). Volcano plots illustrate the distribution of differentially expressed genes across the three comparison groups (Figure 3c–e).

### 2.3. Functional Enrichment Analysis of Differentially Expressed Genes

Gene Ontology (GO) and Kyoto Encyclopedia of Genes and Genomes (KEGG) enrichment analyses (Table S2) were performed to explore the functional characteristics of the upregulated and downregulated DEGs, identifying significantly enriched terms, pathways, and signature genes within specific expression patterns. In the IMAdCs2 vs. CO_IMAdCs2 comparison, upregulated DEGs demonstrated enrichment in 2197 GO terms (255 significant at *p* < 0.05), while downregulated DEGs showed 2160 enriched terms (105 significant). Bubble plots of selected top-ranked terms were generated based on statistical significance (Supplementary Figures S1 and S2). Upregulated genes primarily clustered in lipid metabolism pathways, mitochondrial functions, and molecular binding processes, including fatty acid synthase activity, fatty acid metabolic processes, mitochondrial inner membrane organization, and lipid binding. Downregulated DEGs were predominantly associated with lipid storage regulation, lipid localization, and mitochondrial organization.

KEGG pathway analysis revealed 307 enriched pathways (34 significant) for upregulated genes and 204 pathways (8 significant) for downregulated genes, visualized through categorical annotation diagrams (Supplementary Figures S3 and S4).

**Figure 4:**
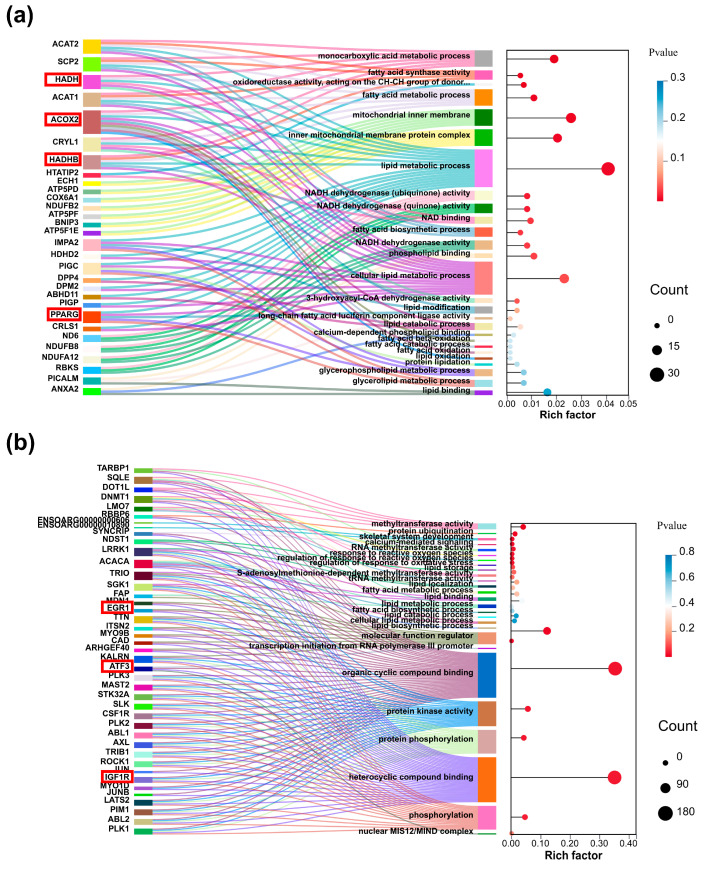
Key entries and gene visualization analysis of GO enrichment results for IMAdCs2 vs. CO_IMAdCs2 groups. (**a**) Sankey bubble plot of up-regulated differentially expressed genes in IMAdCs2 vs. CO_IMAdCs2 group GO enrichment results, where the left color block is the differentially expressed gene and the right is the corresponding GO entry and bubble plot. (**b**) Sankey bubble plot of down-regulated differentially expressed genes in IMAdCs2 vs. CO_IMAdCs2 group GO enrichment results. We highlighted key genes with red boxes to highlight.

**Figure 5:**
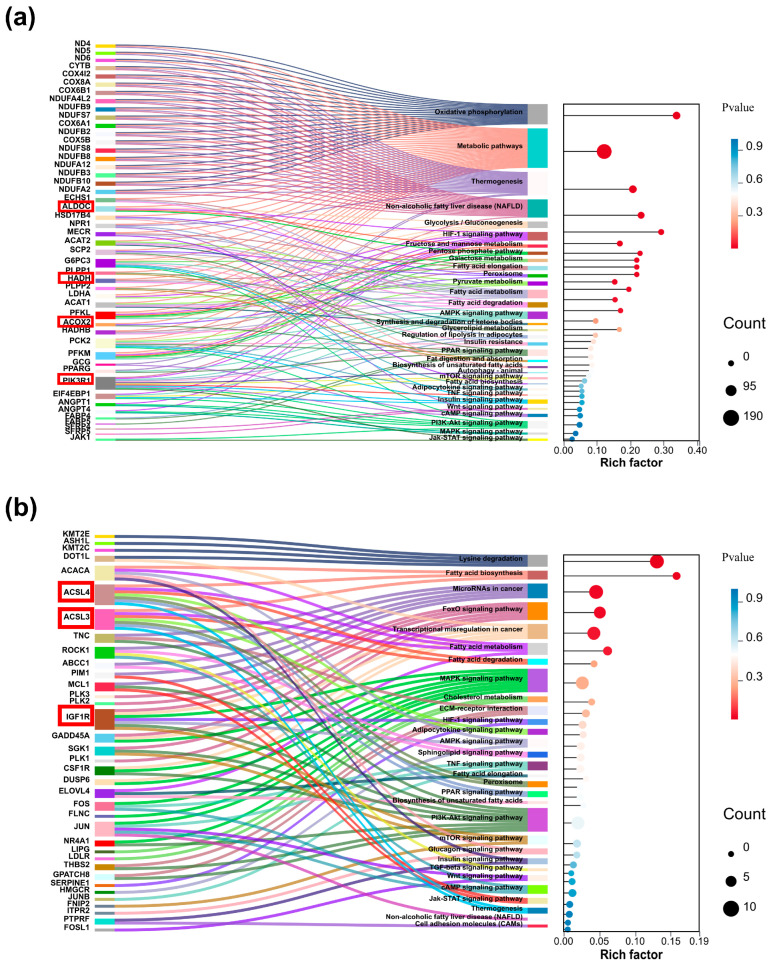
Key entries and gene visualization analysis of KEGG enrichment results for IMAdCs2 vs. CO_IMAdCs2 group. (**a**) Sankey bubble map of up-regulated differentially expressed genes in IMAdCs2 vs. CO_IMAdCs2 group KEGG enrichment results, where the left color block is the differentially expressed gene and the right color block represents the KEGG pathway enriched to and bubble map. (**b**) Sankey bubble map of down-regulated differentially expressed genes in IMAdCs2 vs. CO_IMAdCs2 group KEGG enrichment results. We highlighted key genes with red boxes to highlight.

Complementary analyses of the SMSCs2 vs. CO_SMSCs2 and CO_IMAdCs2 vs. CO_SMSCs2 groups are presented in Supplementary Figure S5 and S6, respectively (Table S2). To elucidate molecular mechanisms underlying co-culture-mediated lipid deposition regulation, key pathways and genes from IMAdCs2 vs. CO_IMAdCs2 analyses were visualized using Sankey bubble diagrams (Figure 4 and Figure 5), highlighting central regulatory networks in intramuscular adipogenesis.

### 2.4. Protein–Protein Interaction (PPI) Network and Module Analysis

Protein–protein interaction (PPI) networks were constructed for both upregulated and downregulated differentially expressed genes (DEGs), followed by module analysis utilizing the k-means clustering algorithm. Three significant subnetworks were identified in each network, represented in red, green, and blue, respectively, and visualized using Cytoscape software (Version number v3.7.1) based on degree values (Supplementary Figures S7 and S8). Furthermore, key genes with high degree values in the upregulated DEGs were selected based on degree values. The key genes identified in the red module included *LDHA*, *PKM*, *ALDOC*, *PPARG*, *PIK3R1*, *PFKL*, *PFKM*, *GCG*, *FYN*, *FABP4*, *FABP5*, and *ANGPT1*; in the green module, key genes included *NDUFS8*, *UQCRB*, *NDUFB8*, *NDUFA2*, *COX6B1*, *UQCR10*, *NDUFB9*, *COX6A1*, *NDUFA12*, *NDUFB3*, *NDUFB10*, *NDUFB6*, *COX4I2*, *NDUFS7*, *ND6*, *ND4*, and *ND5*; and in the blue module, key genes included *ACOX2*, *ECHS1*, *HADHB*, *ACAT1*, and *ACAT2*. In the red module of the downregulated differentially expressed genes, the key genes identified included *ITSN2*, *MYO9B*, *ABL1*, *IGF1R*, and *PLK1*; in the green module, the key genes identified included *EGR1*, *FOS*, *ATF3*, *JUN*, and *JUNB*; and in the blue module, the key genes identified included *ACACA*, *HMGCR*, *ACSL4*, *ACSL3*, and *KMT2E*.

**Figure 6:**
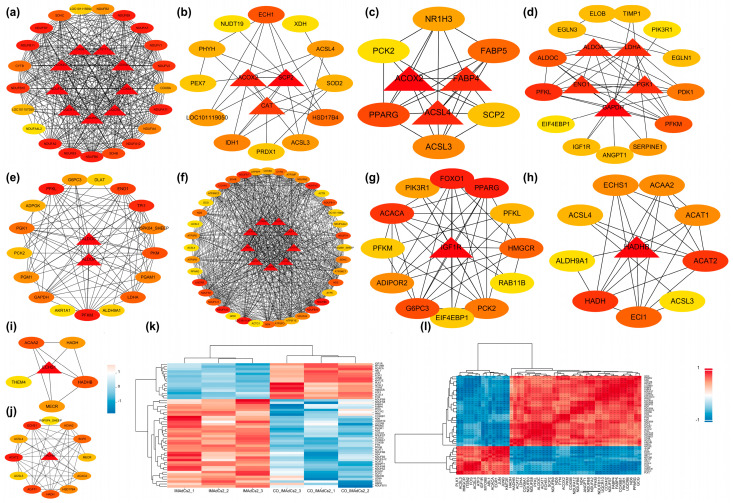
Protein−protein interaction (PPI) network analysis of key KEGG pathways in the IMAdCs2 vs. CO− IMAdCs2 group and identification and analysis of key genes. (**a**–**j**): Protein−protein interaction (PPI) network analysis was conducted for non-alcoholic fatty liver disease (NAFLD), peroxisome, PPAR signaling pathway, HIF−1 signaling pathway, glycolysis/gluconeogenesis, thermogenesis, AMPK signaling pathway, fatty acid degradation, fatty acid elongation, and fatty acid metabolism. (**k**) Hierarchical clustering heatmap of key genes, with colors from blue to red indicating low to high gene expression. (**l**) The heatmap illustrates the correlation of key genes, with color gradients transitioning from light to dark indicating low to high gene correlation magnitudes. Positive correlations are represented in red, while negative correlations are depicted in blue.

Furthermore, ten KEGG pathways exhibiting significant enrichment were selected for hub gene identification, and a protein–protein interaction (PPI) network was constructed to support this analysis. Based on the above analysis, we found that *NDUFB8*, *COX6B1*, *UQCR10*, *COX6A1*, *COX5B*, *NDUFB9*, *NDUFA2*, *NDUFB3*, *ACOX2*, *PPARG*, *PFKL*, *PFKM*, *ALDOC*, *HADHB*, *ACAT2*, *HADH*, *ECHS1*, *SCP2*, *FABP4*, *ACSL4*, *IGF1R*, and *FABP5* are hub genes with darker colors. Interestingly, *ACOX2*, *PPARG*, *ALDOC*, *HADHB*, and *HADH* are at the core of several pathways. We then performed hierarchical clustering and correlation analysis on the key genes selected by the two methods above, and Figure 6k shows the significant differences in gene expression levels across different samples.

### 2.5. Identification of Ligands and Receptors Affected by Co-Culture and Construction of Ligand–Receptor Pairs Mediating Autocrine and Paracrine Crosstalk in Homologous and Heterologous Cell Types

**Figure 7:**
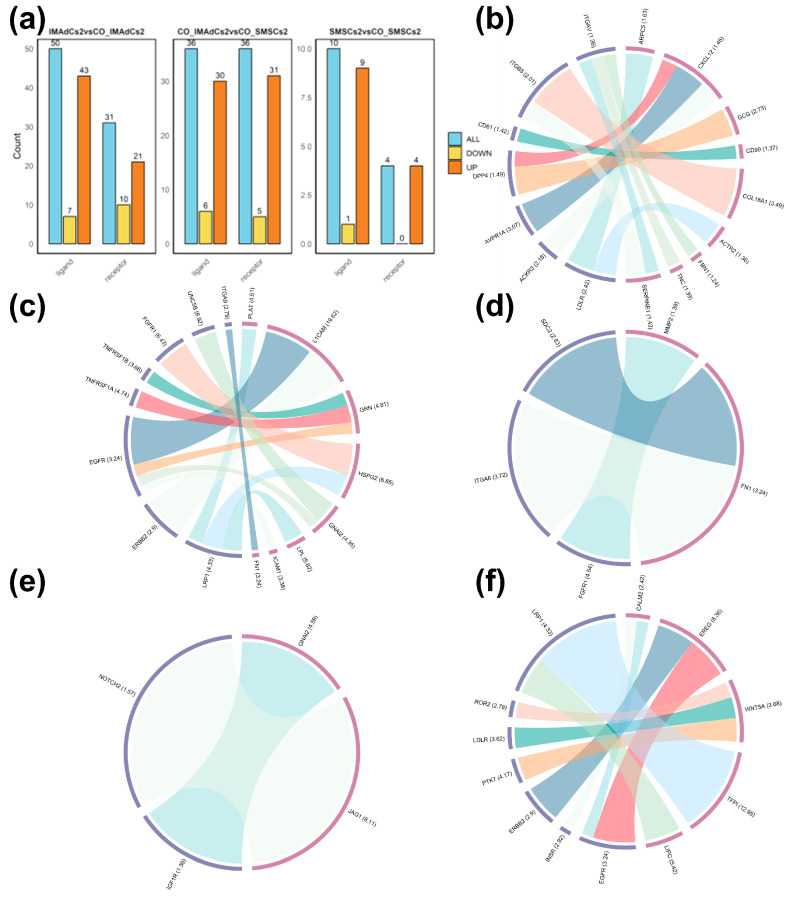
Identification of ligands and receptors affected by co-culture of IMAdCs and SMSCs in Tan sheep, as well as the ligand–receptor pairs mediating autocrine and paracrine signaling-related intercellular communication between IMAdCs and SMSCs. (**a**) A bar chart showing the number of ligands and receptors identified in each group. (**b**) A Circos plot constructed using the differentially expressed ligands and receptors between the IMAdCs2 and CO-IMAdCs2 groups, showing the ligand–receptor pairs mediating autocrine signaling in IMAdCs2 of Tan sheep. (**c**) A Circos plot constructed using upregulated ligands and receptors differentially expressed in the CO-IMAdCs2 vs. CO-SMSCs2 group, showing the ligand–receptor pairs mediating autocrine signaling in IMAdCs2 of Tan sheep. (**d**) Ligand–receptor pairs identified through two methods mediating signaling from IMAdCs to SMSCs. (**e**) Ligand–receptor pairs mediating signaling from SMSCs to IMAdCs, identified by comparing upregulated ligands in the SMSCs2 vs. CO-SMSCs2 group and downregulated receptors in the IMAdCs2 vs. CO-IMAdCs2 group. (**f**) Ligand–receptor pairs mediating signaling from SMSCs to IMAdCs, identified by comparing downregulated ligands and upregulated receptors in the CO-IMAdCs2 vs. CO-SMSCs2 group.

Figure 7a displays the gene counts of differentially expressed ligands and receptors identified across the three combinations. The IMAdCs2 vs. CO-IMAdCs2 group had the highest total number of identified ligands and receptors, with 50 and 31, respectively, while the SMSCs2 vs. CO-SMSCs2 group had the lowest, with 10 and 4, respectively. Using the first method, a total of 12 autocrine ligand–receptor pairs were identified in IMAdCs (Figure 7b), with the top-ranked pairs being COL18A1-ITGB5, GCG-DPP4, and CXCL12-AVPR1A. Additionally, using the second method, a total of 13 autocrine ligand–receptor pairs were identified in IMAdCs (Figure 7c), with the top-ranked pairs being L1CAM-EGFR, L1CAM-ERBB2, and HSPG2-FGFR1. However, neither the first nor the second method detected autocrine pairs in SMSCs. Using the first method, one paracrine ligand–receptor pair, MMP2-FGFR1, was identified from IMAdCs to SMSCs. The second method identified two ligand–receptor pairs, FN1-ITGA6 and FN1-SDC2. These ligand–receptor pairs identified by both methods were combined into a single diagram (Figure 7d). Additionally, regarding paracrine signaling from SMSCs to IMAdCs, using the first method, two ligand–receptor pairs, GNAI2-IGF1R and JAG1-NOTCH2, were identified (Figure 7e). The second method identified nine ligand–receptor pairs, with the top-ranked ones being TFPI-LRP1, EREG- EGFR, and EREG-ERBB2. The corresponding data are presented in Table S3.

### 2.6. qRT-PCR to Verify the Differentially Expressed Genes

Three upregulated genes and three downregulated genes were validated through qRT-PCR based on RNA-Seq results. The analysis confirmed the successful detection of six functional candidate genes (*PPARγ*, *FABP5*, *FYN*, *ATF3*, *EGR1*, and *IGF1R*). The RNA-seq data corroborated the observed gene expression patterns in IMAdCs2 and CO-IMAdCs2 during the late differentiation stage (Supplementary Figure S9).

## 3. Discussion

IMF content is a polygenic characteristic of animals and a significant factor in meat quality. Increased intramuscular fat accumulation can enhance the formation of marbling patterns in meat, thereby improving its taste, flavor, color, and other quality attributes [16,17,18]. Intramuscular fat is very complicated and metabolically active, including several genes and complex metabolic processes and pathways. The regulating mechanism of intramuscular fat deposition, on the other hand, is poorly understood [19]. In this study, transcriptome sequencing was conducted on intramuscular preadipocytes (IMAdCs), co-cultured IMAdCs (CO-IMAdCs), skeletal muscle satellite cells (SMSCs), and co-cultured SMSCs (CO-SMSCs) during the late differentiation stage to elucidate the effects of lamb SMSCs on IMAdCs and their underlying molecular mechanisms. A single/co-culture experimental model of IMAdCs was employed to assess the impact of SMSCs on IMAdC biology. Principal component analysis (PCA), correlation analysis, and hierarchical clustering were used to validate reproducibility within groups and highlight differences between single and co-cultured IMAdCs. Biological replicates demonstrated robust reproducibility, with significant distinctions observed between single and co-cultured groups. Differentially expressed genes (DEGs) were systematically analyzed and categorized, with a focus on KEGG signaling pathways associated with lipid deposition, fatty acid metabolism, and energy metabolism. Notably, most DEGs promoting lipid deposition were downregulated in co-cultured IMAdCs, while DEGs inhibiting lipid deposition were upregulated. Significantly enriched upregulated pathways included oxidative phosphorylation, metabolic pathways, non-alcoholic fatty liver disease (NAFLD), glycolysis/gluconeogenesis, and fatty acid metabolism/degradation. Pathways such as AMPK and PPAR signaling exhibited enrichment without reaching statistical significance. These pathways collectively regulate lipid catabolism, energy homeostasis, and intercellular communication. Downregulated pathways featured lysine degradation, fatty acid biosynthesis, and FoxO signaling, providing critical insights into lipid metabolic regulation. Furthermore, a subset of ligands and receptors significantly influenced by cell co-culture was identified, offering critical insights into the formation of ligand–receptor pairs that mediate intracellular and extracellular interactions between the investigated cell types. These findings demonstrate that co-culture conditions significantly modulate autocrine signaling pathways in IMAdCs and paracrine-mediated cell–cell communication between IMAdCs and SMSCs. These findings significantly advance our understanding of the molecular mechanisms underlying intramuscular fat (IMF) deposition and its regulatory interplay with skeletal muscle satellite cells (SMSCs).

Intramuscular adipogenesis involves multiple genes and intricate metabolic pathways. Results from GO and KEGG enrichment analyses, integrated with protein–protein interaction network analysis, demonstrated that differentially expressed genes play a significant role in lipid deposition and metabolic regulation. The top ten KEGG-enriched pathways demonstrated that key genes including *ACOX2* and *PPARG* occupy central regulatory positions across multiple metabolic pathways, underscoring their pivotal roles in orchestrating essential biochemical processes. In the present study, *PPARG*, *ACOX2*, *PIK3R1*, *FABP5*, *FYN*, *ALDOC*, *PFKM*, *PFKL*, *HADH*, and *HADHB* exhibited downregulation in co-cultured IMAdCs, whereas *ACSL3*, *ACSL4*, *ATF3*, *EGR1*, and *IGF1R* showed upregulation. These findings align with our previous observations regarding lipid droplet quantification patterns [10], reinforcing the consistency of metabolic regulatory mechanisms under experimental conditions.

*PPARγ*, a central regulator of adipogenesis, also activates thermogenic gene transcription in adipose tissue. Its post-translational modifications and dynamic co-regulator recruitment dictate whether adipocytes differentiate toward lipid storage or thermogenesis [20]. By binding to the nuclear receptor coactivator PGC-1α, PPARγ links transcriptional programs to adaptive thermogenesis [21]. As a key transcription factor, it regulates genes encoding adipocyte phenotype-associated proteins [22] and drives adipogenesis by promoting preadipocyte recruitment and adipocyte proliferation [23].

In the peroxisomal signaling pathway, *ACOX2* is significantly upregulated. ACOX, a critical enzyme in fatty acid oxidation [24], suggests *ACOX2* may regulate intramuscular fat deposition in chickens with varying growth rates via the PPAR pathway, given its involvement in multiple fatty acid oxidation steps [25]. The HIF-1 signaling pathway, which is implicated in islet differentiation, adipocyte regulation, and immune function, also plays a significant role. *PIK3R1*, a key player in insulin signaling, was identified as a candidate gene for intramuscular fat deposition in Laiwu pigs, along with *NPY1R* and *NPY5R*, which are involved in the regulation of feeding behavior and fat accumulation [26,27]. Our findings indicate that *PIK3R1* positively regulates intramuscular preadipocyte development.

*FABP5*, a key adipokine, modulates lipid metabolism and transport by binding free fatty acids. It promotes the synthesis of fatty acids into complex lipids, orchestrates intracellular lipid transport, and contributes to processes such as cell cycle progression, migration, and de novo fatty acid synthesis. Additionally, *FABP5* modulates the activity of key enzymes, including *FASN* and *SCD1* [28,29,30]. Activation of the Akt signaling pathway by *FABP5* ensures preadipocyte survival, while its downregulation triggers caspase 3-mediated apoptosis in differentiated preadipocytes [31,32,33].

*FYN*, another pivotal protein, promotes lipid utilization and energy expenditure in knockout mice, elevating fatty acid oxidation in skeletal muscle and adipose tissue while mitigating obesity associated with AMPK activation [34,35,36]. Inhibition of *FYN* activity further elevates energy expenditure and lipid utilization. Our study revealed significantly higher *FABP5* and *FYN* expression in the IMAdCs2 group compared to CO-IMAdCs2, suggesting their synergistic promotion of lipid synthesis and accumulation via the PPAR pathway.

*ALDOC*, a novel target for regulating intramuscular fat deposition, may improve muscle fat infiltration and meat quality through the AKT-mTORC1 signaling pathway [37]. This glycolysis-related gene also participates in cholesterol biosynthesis and regulates cholesterol/triglyceride levels in mice [38]. *PFKM*, a muscle-specific phosphofructokinase, catalyzes the phosphorylation of fructose-6-phosphate and is critically involved in cardiovascular diseases; however, its mechanisms in glycolysis and heart failure warrant further investigation [39].

Under glucose deprivation, *PFKL* undergoes phosphorylation, reducing its enzymatic activity while enhancing its interaction with lipid droplet protein *PLIN2* [40]. Energy stress induces a novel protein kinase function in *PFKL*, mediating lipid droplet-mitochondrial coupling and highlighting its central role in coordinating glycolysis, lipid metabolism, and mitochondrial oxidation [41]. *EGR1* suppresses *PFKL* transcription by binding to its promoter region, thereby inhibiting glycolysis [41]. Figure 6 demonstrates that *PFKL* and *PFKM* are jointly involved in the HIF-1 signaling pathway, glycolysis/gluconeogenesis, and the AMPK signaling pathway. These findings suggest that *PFKL* and *PFKM* may be key genes in suppressing lipid deposition in intramuscular preadipocytes during co-culture with muscle satellite cells, primarily exerting their effects through the aforementioned pathways.

*HADHB*, a fatty acid β-oxidation enzyme, forms a heterodimer with *HADHA* to execute the three enzymatic functions of mitochondrial fatty acid oxidation [42]. Mitochondrial β-oxidation, an evolutionarily conserved pathway, degrades long-chain fatty acids to fulfill cellular energy requirements. The mitochondrial trifunctional protein (MTP), consisting of *HADHA* and *HADHB* subunits, facilitates the three critical enzymatic reactions of this pathway [43]. Short/medium-chain 3-hydroxyacyl-CoA dehydrogenase (SCHAD), encoded by the *HADH* gene, catalyzes the third step of mitochondrial β-oxidation [44]. This enzyme primarily targets medium- and short-chain fatty acids, serving a critical function in thermogenesis, body weight modulation, and insulin secretion in response to nutritional cues. Figure 6 illustrates that *HADH* and *HADHB* are jointly involved in fatty acid degradation, fatty acid elongation, and fatty acid metabolism, occupying central positions within these pathways. This indicates that *HADH* and *HADHB* may serve as key genes in the suppression of lipid deposition in intramuscular preadipocytes during co-culture with muscle satellite cells, primarily exerting their effects through the aforementioned pathways.

Figure 6 reveals that six differentially expressed genes of interest are jointly involved in the PPAR signaling pathway, namely *PPARG*, *ACOX2*, *FABP5*, *FABP4*, *ACSL3*, and *ACSL4*. Among these, *ACOX2*, *FABP4*, and *ACSL4* occupy central positions, suggesting that this pathway may be a key regulatory route for lipid deposition in intramuscular preadipocytes. Additionally, five differentially expressed genes of interest are jointly involved in the HIF-1 signaling pathway, specifically *IGF1R*, *ALDOC*, *PIK3R1*, *PFKM*, and *PFKL*. The PPAR (Peroxisome Proliferator-Activated Receptor) signaling pathway plays a central regulatory role in fat deposition and adipose tissue development, with PPARγ being particularly crucial as it activates various target genes related to adipogenesis and fat storage, promoting the differentiation of preadipocytes into mature adipocytes and enhancing lipid accumulation. Similarly, the HIF-1 (Hypoxia-Inducible Factor 1) signaling pathway also plays a key role in the regulation of fat deposition and associated metabolic diseases, particularly in conditions of local hypoxia within adipose tissue during obesity. In conclusion, both the PPAR and HIF-1 signaling pathways are likely key routes for regulating lipid deposition in intramuscular preadipocytes.

Current research findings suggest that *PPARG*, *ACOX2*, *PIK3R1*, *FABP5*, and *FYN* promote fat deposition, and these genes are positively correlated with *ALDOC*, *PFKM*, *PFKL*, *HADH*, and *HADHB*. Their downregulation in co-cultured IMAdCs indicates that they may play a key regulatory role in the process of SMSC-mediated inhibition of fat deposition in Tan sheep. Notably, *ACOX2*, *PPARG*, *ALDOC*, *HADHB*, and *HADH* occupy central positions in several key pathways. Furthermore, previous studies have shown that when pig intramuscular adipogenic cells are co-cultured with myogenic cells, the myogenic niche limits the differentiation potential of preadipocytes by secreting inhibitory signaling molecules such as *CCL5* [45,46]. Conversely, under high glucose conditions, adipocytes can transfer miRNAs (such as miR-130b) or lipid metabolism-related proteins (such as *FABP4*) via exosomes, promoting lipid accumulation in other cells (such as myocytes or tumor cells) in the co-culture [47,48]. Another study found that intramuscular preadipocytes in chickens promote lipid deposition in muscle satellite cells while inhibiting their differentiation [49]. These findings are consistent with our previous observations of lipid droplet quantification patterns and the transcriptomic sequencing results of this study [10]. Therefore, these 10 genes may be key regulators of lipid deposition in IMAdCs2 under SMSCs2 regulation, providing new insights into the molecular mechanisms underlying the interactions between Tan sheep muscle satellite cells and intramuscular adipocytes.

We observed upregulation of *ACSL3*, *ACSL4*, *ATF3*, *EGR1*, and *IGF1R* in co-cultured IMAdCs, suggesting their potential roles as key regulators in SMSC-mediated suppression of lipid deposition in Tan sheep. *ACSL3* maintains lipid homeostasis in adipocytes by regulating transcription factor activity [50] and is implicated in the differentiation of 3T3-L1 adipocytes in mice. *ACSL4* catalyzes the CoA activation of fatty acids to generate acyl-CoA [51], playing pivotal roles in intracellular lipid storage, cholesterol mitochondrial transport, and arachidonic acid metabolism [52]. Polymorphisms in the *ACSL4* gene exhibit a strong correlation with intramuscular fat content and fatty acid composition across various pig breeds, with its mRNA abundance reaching peak levels during mid-stage adipocyte differentiation and remaining elevated throughout the process [53]. In the IMAdCs group, *PPARγ* was significantly upregulated, while *ACSL3/4* expression was suppressed in the thermogenic signaling pathway during late-stage adipocyte differentiation. This suggests that *PPARγ-ACSL3/4* may regulate lipid differentiation and reduce intracellular lipid deposition through thermogenic signaling in the co-culture system. Additionally, Figure 6 shows that *ACSL3* and *ACSL4* are jointly involved in peroxisomes, the PPAR signaling pathway, thermogenesis, fatty acid degradation, and fatty acid metabolism. This indicates that *ACSL3* and *ACSL4* may be key genes in the inhibition of lipid deposition in intramuscular preadipocytes during the co-culture with muscle satellite cells, primarily exerting their effects through the aforementioned pathways.

Deficiency in *ATF3* exacerbates obesity and metabolic dysregulation in mice subjected to a high-fat diet. Overexpression of *ATF3* inhibits lipogenic genes such as *ChREBP* and *SCD1* while upregulating genes involved in lipolysis and adipose tissue browning [54,55,56]. The significant upregulation of *ATF3* in CO-IMAdCs2 suppresses adipogenesis, consistent with our previous findings [10]. Additionally, mesenchymal stem cell overexpression of *EGR1* inhibits white adipose tissue browning, increases energy expenditure, and mitigates obesity-related metabolic abnormalities [57,58,59]. *EGR1* is known to suppress lipogenesis, and its downregulation, along with *ATF3*, is observed in pigs with high intramuscular fat content [60,61]. These findings suggest that *ATF3* and *EGR1* likely inhibit adipogenesis, aligning with our current results.

The PI3K/AKT-mediated *IGF1R* signaling pathway is critical for skeletal muscle growth and intramuscular fat deposition [62]. Conditional knockout of *IGF1R* in the epididymal fat pad of mice increases adipose tissue mass through enhanced lipogenesis [63]. Simultaneous inhibition of the insulin receptor and *IGF1R* in lipodystrophic mice leads to intracellular accumulation of triglycerides [63]. In the IMAdC group, *PIK3R1* in the HIF-1 pathway was significantly upregulated, while *IGF-1R* was downregulated, underscoring the complex regulatory network governing lipid metabolism. In addition, Figure 6 shows that *IGF1R* and *PIK3R1* are jointly involved in the HIF-1 and AMPK signaling pathways, serving as core genes in both. This suggests that *IGF1R* and *PIK3R1* may primarily inhibit lipid deposition in co-cultured intramuscular preadipocytes through these pathways.

Correlation analysis revealed that energy metabolism-related genes demonstrated significant associations (Figure 6l), such as the NDUFS and COX families, form co-expression modules in the samples, suggesting a potential key role of mitochondrial energy metabolism in fat storage. Meanwhile, the lower correlation between certain genes suggests that they may play roles in different metabolic pathways or stages of fat deposition. Genes such as *NDUFS8*, *NDUFB8*, *NDUFA2*, *NDUFB9*, *NDUFA12*, *NDUFB3*, *NDUFB10*, *NDUFB6*, and *NDUFS7* play crucial roles in mitochondrial membrane transport and the function of NADH dehydrogenase (including NDUFA/B/S subunits). These genes are central to energy metabolism, electron transport, and oxidative phosphorylation [64,65]. Studies have confirmed that metabolic pathways related to adipogenesis are critical for the development of ovine tail adipose tissue, particularly at embryonic day 70, when mitochondrial components are significantly upregulated [66]. Based on these findings, it is hypothesized that mitochondrial energy metabolism plays a key role in fat storage. Results demonstrate that co-culturing skeletal muscle satellite cells with intramuscular preadipocytes inhibits the latter’s development and lipid droplet accumulation.

Our results indicate that co-culturing significantly influences both the autocrine signaling pathways within IMAdCs and the paracrine-mediated communication between IMAdCs and SMSCs. Specifically, L1CAM-EGFR, L1CAM-ERBB2, PLAT-LRP1, LPL-LRP1, and HSPG2-LRP1 are the predicted ligand–receptor pairs involved in autocrine signaling among IMAdCs.

*EGFR* and *ERBB2*, members of the receptor tyrosine kinase (RTK) family, are pivotal in driving lipogenesis through the PI3K-AKT-mTOR and MAPK/ERK signaling pathways. *L1CAM*, a transmembrane adhesion molecule, may interact with *ERBB2* or *EGFR* through its intracellular domain, potentially enhancing ligand-independent activation. This interaction could lead to continuous activation of downstream AKT or ERK pathways by facilitating receptor dimerization or phosphorylation [67], thereby upregulating genes related to lipogenesis. However, direct binding of *L1CAM* to *ERBB2* or *EGFR* has not been explicitly confirmed. Further studies integrating molecular interaction assays and metabolic analyses are necessary to verify this interaction and elucidate the structural characteristics of these complexes, as well as their downstream signaling differences [68].

*LRP1*, a multifunctional receptor, interacts with diverse ligands and serves a critical role in lipid transport and signal transduction processes. In the early stages of obesity, ligand–receptor interactions involving *LPL*, *LRP1*, and *ApoE* in ATM contribute to lipid signaling, which regulates inflammation and shapes the metabolic microenvironment of adipose tissue [69]. In adipocytes, the expression levels of *LPL* and *LRP1* are critically associated with lipid uptake and storage. Previous studies have demonstrated that *PLAT* interacts with *LRP1*, with *LRP1* facilitating the internalization of *PLAT*; however, the specific molecular mechanisms governing this interaction remain incompletely understood and warrant further exploration [70]. Similarly, *HSPG2* may influence lipoprotein lipase (LPL) activity through its interaction with *LRP1*, thereby affecting the uptake and storage of free fatty acids (FFAs) [70,71].

Our analysis has revealed over a dozen ligand–receptor pairs that facilitate paracrine signaling between IMAdCs and SMSCs, including TFPI-LRP1, EREG-EGFR, EREG-ERBB2, WNT5A-PTK7, WNT5A-LDLR, and WNT5A-ROR2. These interactions play key roles in adipocyte differentiation, lipid metabolism, and extracellular matrix (ECM) regulation.

*LRP1* influences adipocyte differentiation by modulating the Hedgehog pathway, particularly through its interaction with *HPI-4* [72]. Meanwhile, *TFPI* may contribute to the stability of the pericellular microenvironment by inhibiting the uPA/uPAR system, which is associated with fibrinolysis and ECM degradation [72]. Together, these factors help maintain the dynamic balance of fat deposition.

Epiregulin (*EREG*), a ligand of the epidermal growth factor receptor (*EGFR*), is instrumental in modulating glucose uptake and metabolic processes. Studies have shown that in leptin-deficient conditions, *EREG* controls glucose uptake in mice by activating the leptin receptor (LepR), highlighting its significance in metabolic regulation [73]. As a member of the epidermal growth factor (*EGF*) family, *EREG* primarily activates *EGFR* (*ERBB1*) and *ERBB4. ERBB2*, though lacking intrinsic ligand-binding ability, functions as a co-receptor, forming heterodimers with other *ERBB* family members (e.g., *EGFR* or *ERBB4*) to enhance downstream signaling [74]. Notably, ERBB2-ERBB4 heterodimerization significantly increases EREG’s activation sensitivity toward *ERBB4* [75,76]. The EREG-ERBB2 signaling pathway likely involves the MAPK/ERK and PI3K/AKT pathways. While the majority of current research emphasizes its role in driving cell proliferation in malignancies such as breast, gastric, and colorectal cancers [77,78], its specific function in adipose metabolism remains largely unexplored.

*WNT5A*, acting through *ROR2*, regulates cell polarity and motility via the *RHOA*, *JNK*, and *PKC* signaling pathways [79,80]. In adipose tissue, this pathway may influence adipocyte arrangement and intercellular adhesion, thereby affecting fat distribution [81]. Additionally, *WNT5A* may exert paracrine effects on cells expressing *LDLR*, such as hepatocytes or adipocytes, to regulate lipid uptake and storage-related genes [79]. *LDLR* may regulate the cell membrane microenvironment, such as lipid raft organization, to influence the stability of WNT5A-PTK7/ROR2 receptor complexes, thereby indirectly modulating adipocyte metabolic processes [79,81].

*PTK7*, a catalytically inactive tyrosine kinase receptor, regulates cell polarity by binding to non-canonical WNT signaling molecules such as *WNT5A*. Studies indicate that *PTK7* forms heterodimeric complexes with *ROR2*, enabling a coordinated response to WNT5A signaling [82,83]. The WNT5A-PTK7/ROR2 signaling axis indirectly affects fat deposition and the metabolic microenvironment by regulating cell migration, polarity, and inflammatory responses. However, the potential association between *WNT5A* and *LDLR* remains to be validated. Future research integrating single-cell sequencing and metabolomics is necessary to clarify the precise roles of these pathways in adipose tissue heterogeneity and metabolic reprogramming [81,83].

In summary, the expression of the *PPAR*, *ACOX2*, *PIK3R1*, *FABP5*, *FYN*, *ALDOC*, *PFKM*, *PFKL*, *HADH*, and *HADHB* genes was dramatically down-regulated in CO-IMAdCs2, while the *ACSL3*, *ACSL4*, *ATF3*, *EGR1*, and *IGF1R* genes were significantly up-regulated. We conclude that in the Tan sheep co-culture system, skeletal muscle satellite cells impede intramuscular preadipocyte development and enhance intramuscular fat metabolism. Skeletal muscle satellite cells have been identified as key regulators in the development of intramuscular preadipocytes; however, the specific mechanisms underlying this regulation require further investigation. Such research should clarify the influence and regulatory mechanism of Tan sheep skeletal muscle satellite cells on the adipogenic differentiation of intramuscular preadipocytes, and it should provide a theoretical foundation for future research on changes in the structure and function of intramuscular preadipocyte differentiation-related genes, as well as the regulation of signal transduction pathways, in order to improve meat quality and increase Tan sheep economic value.

## 4. Materials and Methods

### 4.1. Culture and Differentiation Identification of Intramuscular Preadipocytes and Skeletal Muscle Satellite Cells

Three 14-day-old healthy and disease-free Tan sheep were chosen (courtesy of the Professional Cooperative of Chunhao Lincao Industry in Yanchi County, Ningxia). Longissimus dorsi muscle tissue was aseptically harvested post euthanasia and preserved in sterile phosphate-buffered saline (PBS) supplemented with a dual antibiotic solution (100 IU/mL Penicillin–Streptomycin) for subsequent use. The muscle was divided into tissue blocks around 1 mm^3^ in size. Next, 0.2% type II collagenase was added, and the mixture was placed in a 37 °C water bath for 1.5 h, shaking once every 5 min. To end digestion, an identical volume of complete medium (DMEM/F12 + 10% FBS + 100 IU/mL penicillin) was added. The digests were passed through cell screens with 200-mesh and 400-mesh pore diameters, and the filtrate was centrifuged at 1500 r/min for 10 min before discarding the supernatant. The cells were grown in a CO_2_ incubator for 3 to 5 days after being resuspended in full media. When the primary cell culture reached about 80% confluence, 0.25% trypsin was used to digest the sample for 3 min, followed by the addition of two volumes of trypsin complete media to stop the digestion and then subculturing. When the second generation of cells reached 80% confluence, oriCellSD rat adipogenic stem cell adipogenic differentiation medium A liquid was applied for 72 h, followed by OriCellSD rat adipogenic stem cell lipolytic induction differentiation medium B solution for 24 h to induce differentiation. On day 8, intramuscular preadipocyte differentiation was triggered, and the cells were identified by Oil Red O staining. Following adipogenic induction and maturation of IMAdCs, the medium was completely removed, and the cells were rinsed twice with PBS. Subsequently, the cells were fixed using 4% paraformaldehyde for 30 min, washed twice with PBS, and stained with Oil Red O for 60 min. The staining solution was discarded, followed by two PBS washes and examination under an inverted microscope. The extraction was carried out in 60% isopropanol, and the OD value of the extract at 510 nm was measured.

The remaining tissue blocks were first digested in a 37 °C water bath for 50 min with 0.1% collagenase type I. The tissues were then digested with 0.25% trypsin (Gibco, Thermo Fisher Scientific, Waltham, MA, USA) for 10 to 20 min (shaking once every 10 min), and an equal volume of complete medium (DMEM/F12 + 10% FBS + 10% horse serum + 100 IU/mL penicillin) was added to terminate digestion after complete digestion. To extract SMSCs, the digestion solution was passed through a cell screen with a particle size of 200 targets, the filtrate was centrifuged at 1000 r/min for 10 min, and the supernatant was discarded. The cells were grown in a CO_2_ incubator for 3 to 5 days after being resuspended in full media. When the primary cell culture reached 80% to 90% confluence, the culture medium was aspirated, cleaned three times with PBS, and digested for three minutes with 0.25% trypsin, and then we stopped the digestion with an equal volume of full culture solution for subculturing. Myogenic differentiation was induced in the second generation of cells after they had reached 80% confluence in culture. Myogenesis media (98% DMEM/F12+2% horse serum) was added, and after 4 days of myogenic differentiation, the MyHC marker protein of SMSCs was detected using immunofluorescence. SMSCs were seeded into 6-well plates and differentiated for 4 days as described above. The medium was then removed and fixed in 4% paraformaldehyde for 15 min at room temperature before being rinsed three times with PBS. The cells were rinsed three times with PBS before being blocked for one hour with 10% goat serum blocking solution. The cells were rinsed three times with PBS before being treated with primary anti-MyHC antibody (diluted 1:100) overnight at 4 °C. The cells were rinsed three times with PBS before being incubated for one hour at room temperature with FITC-labeled goat anti-rabbit secondary antibody (1:100 dilution). PBS was used to wash the cells three times.

### 4.2. Co-Culture of Intramuscular Preadipocytes and Skeletal Muscle Satellite Cells

Two distinct cell co-culture systems were established utilizing a transwell chamber co-culture plate configuration. Intramuscular preadipocytes were plated at a density of 2.0 × 10^4^ cells/cm^2^ in the upper compartment, while skeletal muscle satellite cells were seeded at a density of 1.0 × 10^5^ cells/cm^2^ in the lower compartment. Myogenic differentiation medium was introduced to the bottom compartment, and adipogenic differentiation medium was supplied to the top compartment when the confluence of skeletal muscle satellite cells and intramuscular preadipocytes reached 90%. Every 48 h, the medium was replaced until lipid droplets and myotubes developed. For transcriptome sequencing, we selected samples comprising co-cultures of intramuscular preadipocytes cultured independently, skeletal muscle satellite cells cultured alone, and intramuscular preadipocytes differentiated on day 8.

### 4.3. Cell Total RNA Extraction and Detection

Total RNA was isolated from IMAdCs2, CO-IMAdCs2, SMSCs2, and CO-SMSCs2 using the TaKaRa MiniBEST Universal RNA Extraction Kit (TaKaRa Bio Inc., Kusatsu, Japan), and RNA purity (OD260/280, OD260/230) was assessed using a Nanodrop spectrophotometer (Thermo Fisher Scientific, Waltham, MA, USA).

### 4.4. Library Construction and High-Throughput Sequencing

Magnetic beads containing oligo (dT) were utilized to enrich mRNA, which was then broken down into short fragments. After that, the mRNA was employed as a template for reverse transcription into cDNA. To create a cDNA library, a certain fragment size of double-stranded cDNA was chosen, and PCR amplification was conducted. The created library was found to meet the standard of a high-quality library in terms of quality (the effective concentration of the library was >4 nM). The cDNA library was sequenced using Illumina high-throughput sequencing technology (hiseQTM255/4000, Illumina Inc., San Diego, CA, USA), and the original sequence information of the fragments to be tested was retrieved.

### 4.5. Sequencing Data Processing

Trimmomatic software (v0.33) was used to eliminate adapter-containing reads in order to assure the accuracy of future analysis results and tightly control the quality of sequencing data. Readings having more than 10% N (N signifies the base information could not be obtained) were eliminated; low-quality readings (quality value Q20 base counts accounting for more than 50% of the reads) were also removed. Illumina Umina HiSeqTM2500/4000 Hi (Illumina Inc., San Diego, CA, USA), the formula for calculating the base quality value with Q phred is as follows: phred Q = −10 log10 €. When measuring the sequence with Tophat software (http://tophat.cbcb.umd.edu/webcite, accessed on 15 April 2023), the sheep genome oviAri3 (http://genome.ucsc.edu accessed on 15 April 2023) should be selected instead of the default parameters. The clean data’s GC, Q20, and Q30 contents were then computed. Finally, using STAR (Version number v2.5.2b) [84], the sequences were matched to the reference genome.

### 4.6. Quantitative Analysis

Gene expression levels were normalized using FPKM to ensure comparability across samples. Expression values were transformed using log10 (FPKM + 1) for visualization, highlighting trends in expression changes among samples. Violin plots (Figure 2a) illustrated the distribution of gene expression across sample groups, with the curve shapes reflecting overall expression patterns and differences in abundance. Principal component analysis (PCA) was conducted to reduce data dimensionality and identify variations between samples (Figure 2b,c). Pearson correlation analysis evaluated the consistency of biological replicates (Figure 2d), while hierarchical clustering depicted the similarities among samples (Figure 2e).

### 4.7. Screening of Differentially Expressed Genes

DESeq2 [15] was utilized to compare the clean data between IMAdCs2, CO-IMAdCs2, SMSCs2, and CO-SMSCs2. Each sample had three biological replicates. Significantly differentially expressed genes were identified using a threshold of |Log2 fold change| > 1 and the Benjamini–Hochberg method with *p* < 0.05. A volcano plot generated in Sangerbox (http://sangerbox.com/home.html, Accessed 21 December 2024) [85] was employed to visualize these differentially expressed genes, followed by functional annotation of the candidate genes.

### 4.8. GO Function and KEGG Pathway Enrichment Analysis of Differentially Expressed Genes

ClusterProfiler (Version 4.4.4) was utilized to perform Gene Ontology (GO) functional enrichment analysis of differentially expressed genes and to assess the statistical enrichment of these genes in Kyoto Encyclopedia of Genes and Genomes (KEGG) pathways [86,87]. A corrected *p* value of <0.05 was established as the significance threshold for identifying differentially expressed genes associated with GO terms and KEGG pathways.

### 4.9. mRNA-Protein Network Interaction Analysis in Adipose Tissue

The genes involved were chosen using information from the GO and KEGG annotations, as well as information from adipogenic pathways. Subsequently, a network diagram was constructed using Cytoscape software (Version number v3.7.1) to identify genes occupying pivotal node positions within the network and to investigate their specific functions and roles in adipose tissue development [88].

### 4.10. Identification of Ligands and Receptors Affected by Co-Culture and Construction of Ligand–Receptor Pairs Mediating Autocrine and Paracrine Crosstalk in Homologous and Heterologous Cell Types

To investigate the molecular pathways underlying intercellular crosstalk, we systematically screened ligands and receptors exhibiting differential expression within the DEGs of each experimental group. Ligand and receptor lists (human) were downloaded from the CellTalk Database (http://tcm.zju.edu.cn/celltalkdb/; accessed on 12 December 2024). Differentially expressed ligands and receptors were systematically identified across three sample sets, and ligand–receptor pairs mediating autocrine and paracrine signaling mechanisms were established to characterize intercellular communication in these cells. To establish molecular bridges, two methodologies were employed to quantify ligand–receptor interaction dynamics, wherein communication scores (CSs) were calculated by multiplying the fold change (FC) values of corresponding ligands and receptors. The first methodological approach entailed comparative analysis of ligands and receptors between mono-cultured and co-cultured cells, whereas the second method employed differentially expressed genes (DEGs) identified in CO-IMAdCs2 versus CO-SMSCs2 as analytical inputs.

### 4.11. qRT-PCR Verification

Based on the results of GO word and KEGG pathway enrichment, three lipid-related functional candidate genes with up-regulated expression in CO-IMAdCs2 cells and three with down-regulated expression in them were chosen. *GAPDH* was utilized as a control gene, and qRT-PCR primers were generated using Primer Premier 5.0 and NCBI software (https://www.ncbi.nlm.nih.gov, accessed on 25 April 2023) using gene sequences from GenBank (https://www.ncbi.nlm.nih.gov/genbank, accessed on 25 April 2023) (Table 1). Quantitative real-time PCR was used to assess the relative mRNA expression levels of IMAdCs2 and CO-IMAdCs2 during adipogenic development. Following total RNA extraction with an RNA extraction kit (TaKaRa MiniBEST Universal RNA Extraction Kit, TaKaRa Bio Inc., Kusatsu, Japan), RNA was reverse transcribed into cDNA with the PrimeScript^TM^ RT Reagent Kit and gDNA Eraser (Perfect Real Time) Reverse Transcription Kit (TaKaRa Bio Inc., Kusatsu, Japan). The PCR reaction system contained 12.5 μL of qPCR mix, 1 μL of upstream and downstream primers, 2 μL of template cDNA, and lastly, the ddH_2_O complement system in a 20 μL volume. The PCR technique was as follows: 3 min of predegeneration at 95 °C; 39 cycles of denaturation at 95 °C for 15 s, annealing for 30 s, and extension at 72 °C for 30 s; and a melting curve analysis.

### 4.12. Statistical Analysis

Each sample was put up in threes. The mRNA content was standardized using the internal reference gene (*GAPDH*), the average value was represented as means ± SD, and the target gene’s relative expression level was computed using the 2^−ΔΔCt^ technique. For statistical analysis, GraphPad Prism 5 was utilized. For comparisons between many groups, one-way ANOVA was employed, while the *t*-test was utilized for comparisons between two groups. The level of significance was set at *p* < 0.05.

## 5. Conclusions

Our transcriptome analysis indicates that skeletal muscle satellite cells regulate the differentiation and lipid metabolism of intramuscular preadipocytes in Tan sheep by modulating key lipid metabolism genes. The upregulated genes (such as *PPARG*, *ACOX2*, *PIK3R1*, *FABP5*, *FYN*, *ALDOC*, *PFKM*, *PFKL*, *HADH*, and *HADHB*) and downregulated genes (such as *ACSL3*, *ACSL4*, *ATF3*, *EGR1*, and *IGF1R*) collectively suggest that skeletal muscle satellite cells can simultaneously inhibit the differentiation of intramuscular preadipocytes and promote intramuscular lipid metabolism. These findings lay a solid foundation for future studies to validate these regulatory mechanisms and explore novel interventions for modulating intramuscular fat accumulation in livestock.

## Figures and Tables

**Table 1:** Related information about the primers used for real-time RT-PCR.

	Up/Down	Log_2_ FC	Primer Sequence (5′ → 3′)
***PPARγ***	up	1.0485	F: TTCCGTTCCCAAGAGCTGAC
			R: CGTGCACGCCGTATTTTAGG
***FABP5***	up	1.2384	F: GGAAGATGGCGCTTAGTGGA
			R: TGGCCATCGCACCTACTTTT
***FYN***	up	0.9934	F: AGACGTGGAAAAAGACCAGTCC
			R: ACTTCCCCAAACTGCCCATT
***EGR1***	down	−2.4717	F: AGTGGAGACGAGTTACCCCA
			R: CCGCTGACCAGACTGAAGAG
***ATF3***	down	−3.0771	F: CACAGTCGCACGCAGAGA
			R: GCTCTGCAATGTTCCCTCCT
***IGF1R***	down	−0.4761	F: ATCCGGATCGAGAAGAACGC
			R: CGTAGTAGTAGTGGCGGCAG
***GAPDH***	/	/	F: AAGCTCATTTCCTGGTACGACA
			R: GGAGGTCGGGAGATTCTCAG

## Data Availability

The data presented in this study are available on request from the corresponding author.

## Supplementary Materials

The following supporting information can be downloaded at: https://www.mdpi.com/article/10.3390/ijms26073414/s1.
