# Supplementary material for: Transcriptomic Analysis of Muscle Satellite Cell Regulation on Intramuscular Preadipocyte Differentiation in Tan Sheep
Source: Int J Mol Sci. 2025 Apr 5;26(7):3414. doi: 10.3390/ijms26073414 (PMC11989785; doi:10.3390/ijms26073414)
Supplement: Supplementary file 1 [file ijms-26-03414-s001.zip › Supplementary1 Figure.docx]

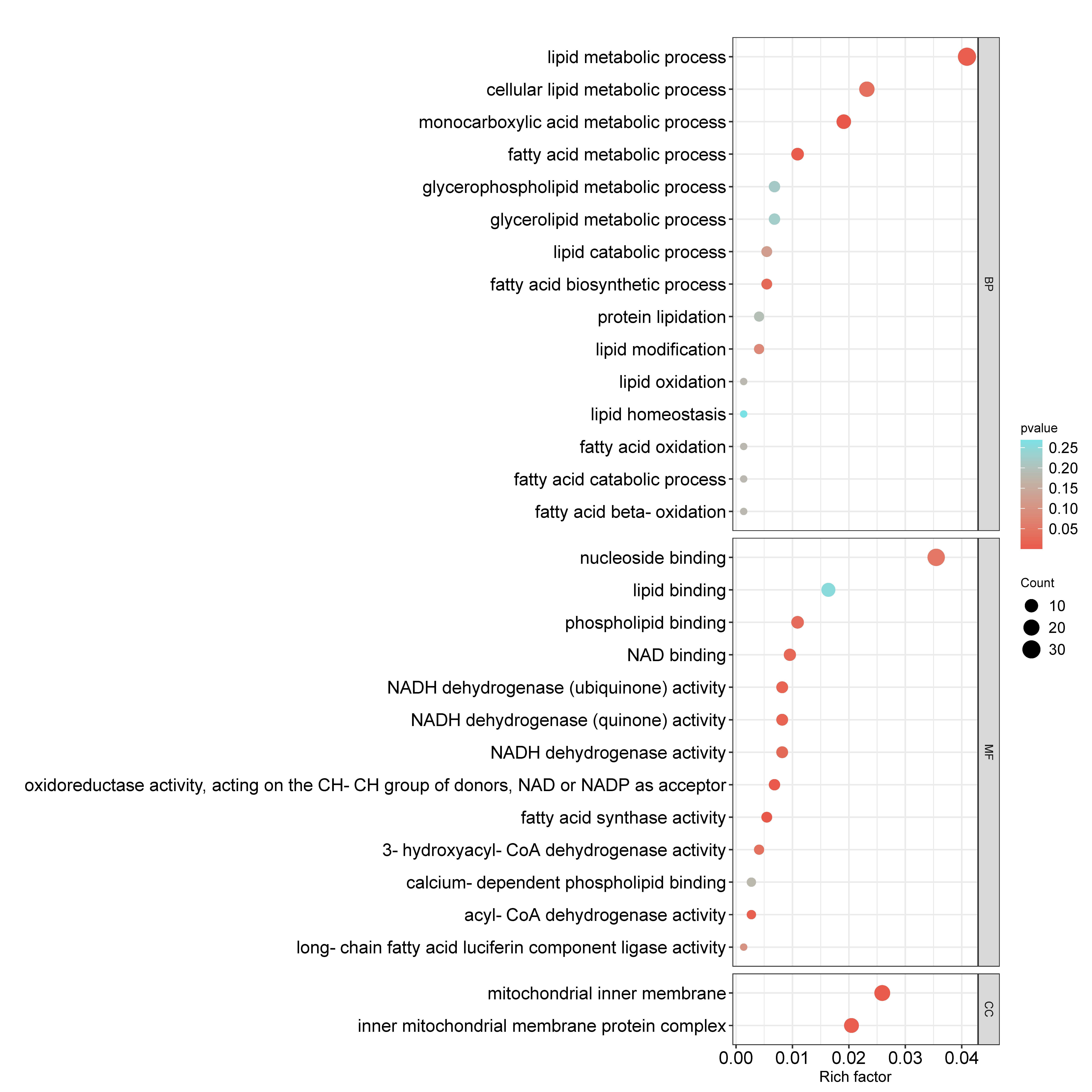


**Supplementary Figure S1.** Bubble plot of GO functional enrichment of up-regulated differentially expressed genes in the IMAdCs2 vs. CO_IMAdCs2 group.


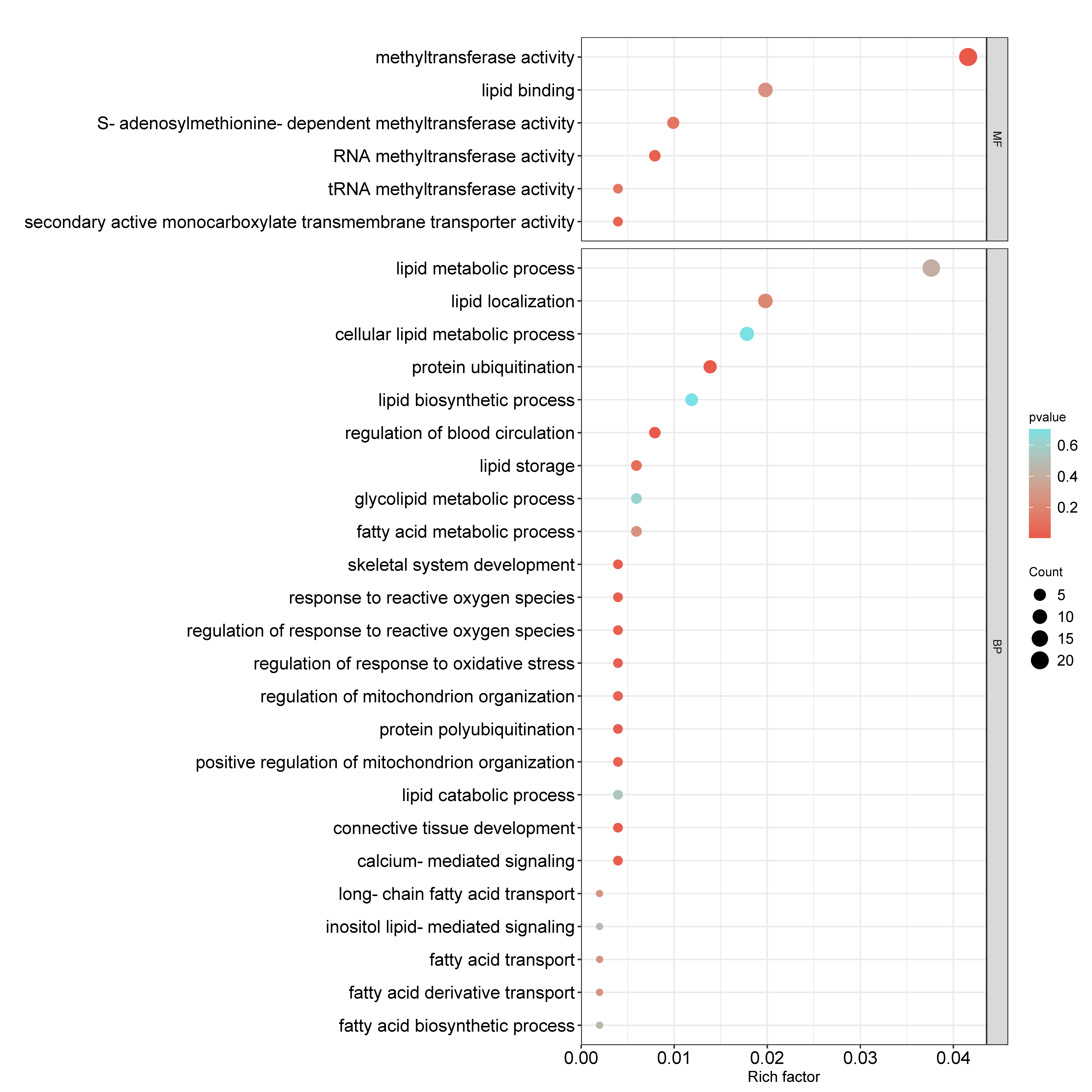


**Supplementary Figure S2**. Bubble plot of GO functional enrichment of down-regulated differentially expressed genes in the IMAdCs2 vs. CO_IMAdCs2 group.


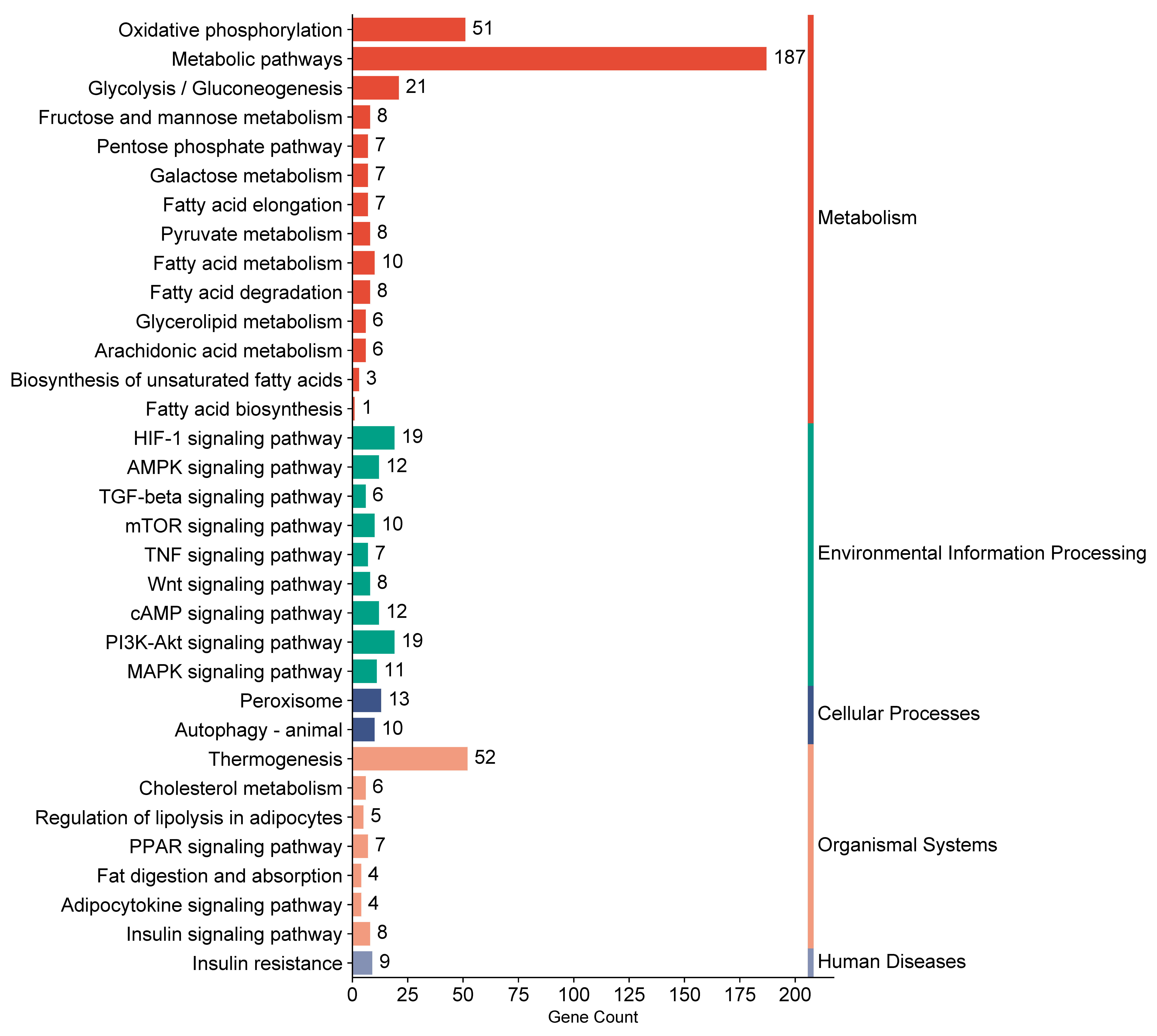


**Supplementary Figure S3**. KEGG functionally enriched categorical annotation map of differentially expressed genes up-regulated in the IMAdCs2 vs. CO_IMAdCs2 group.


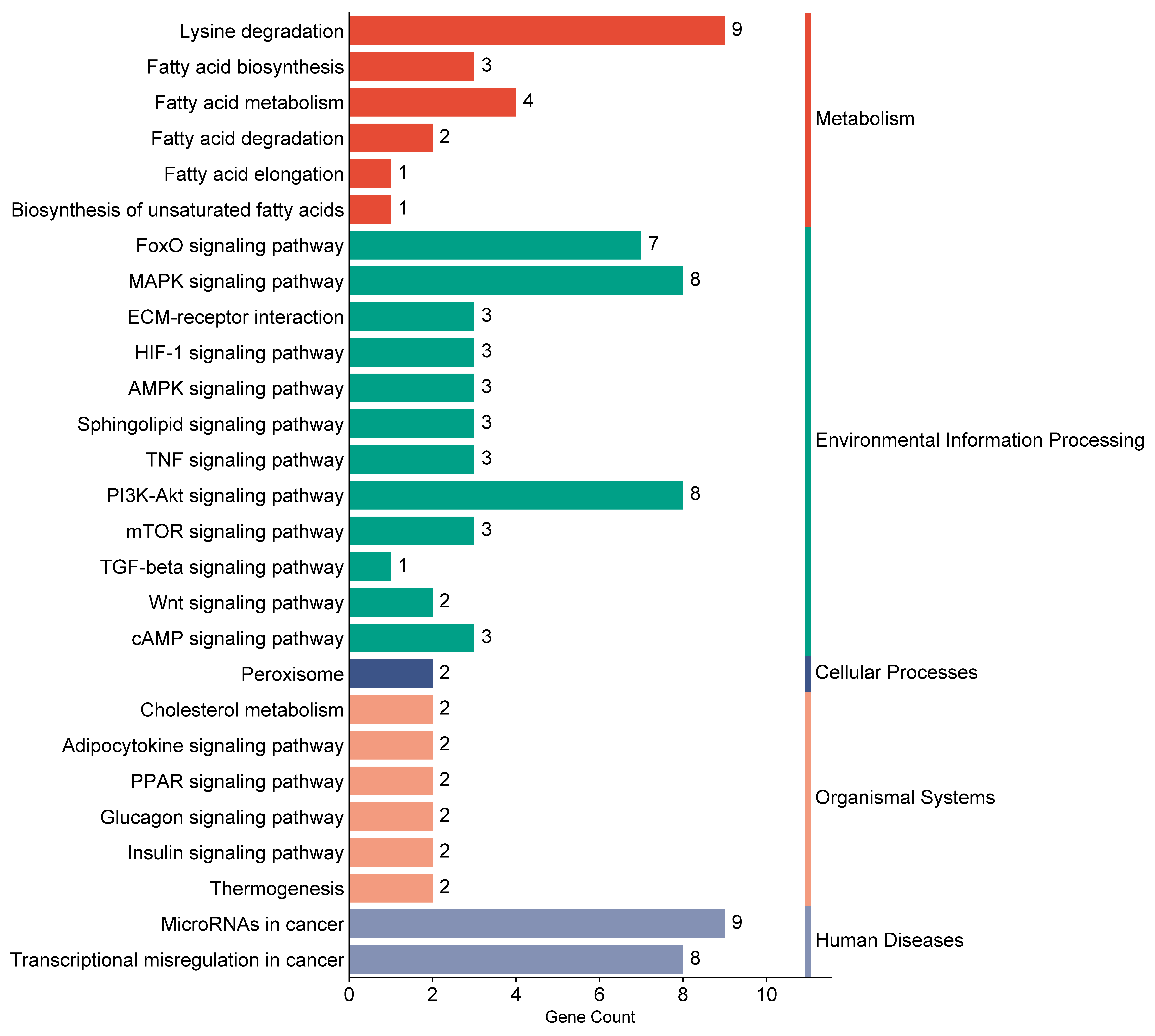


**Supplementary Figure S4**. KEGG functionally enriched categorical annotation map of differentially expressed genes down-regulated in the IMAdCs2 vs. CO_IMAdCs2 group.


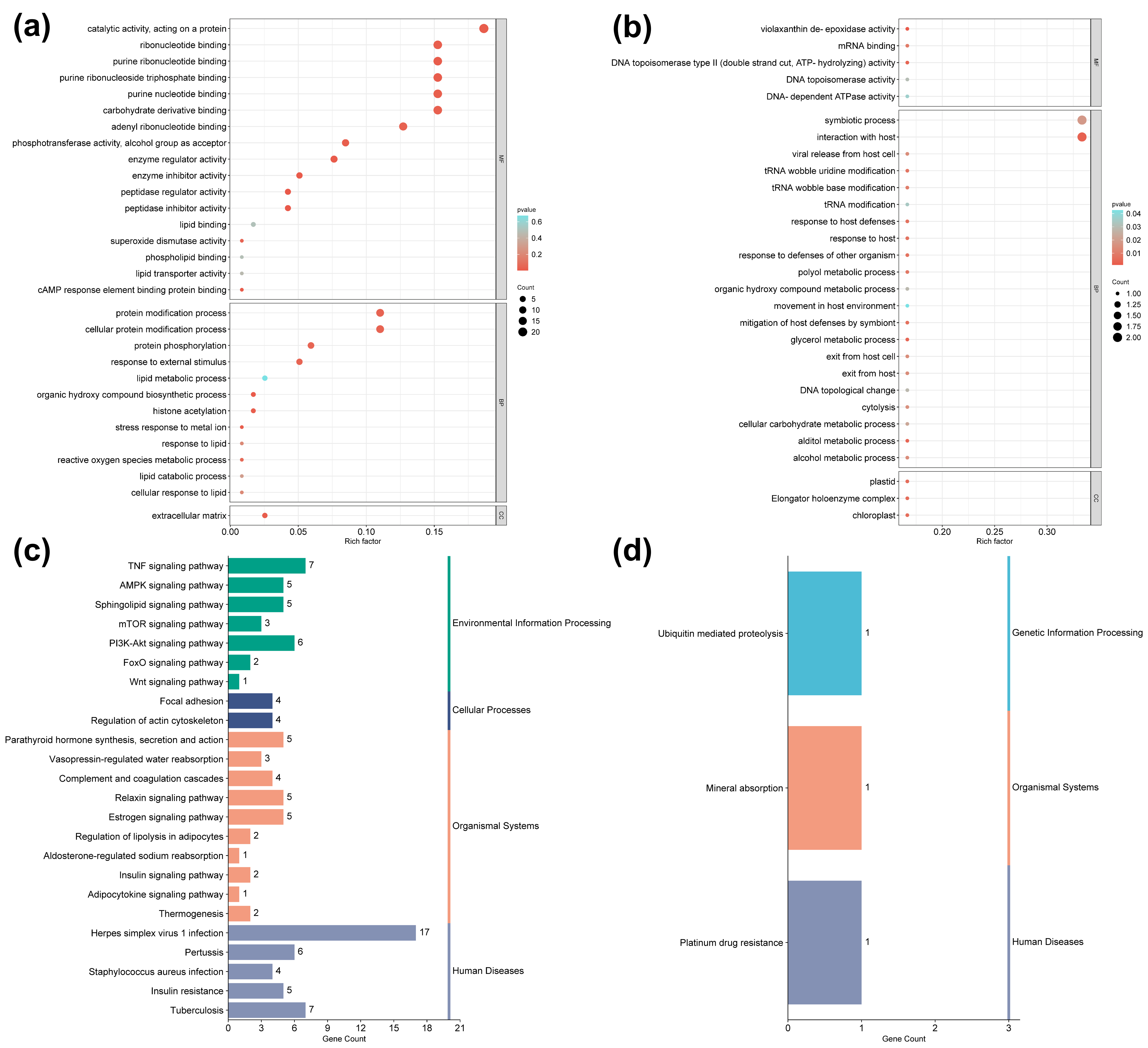


**Supplementary Figure S5.** Functional enrichment analysis of SMSCs2 vs CO_SMSCs2 group. (**a**) GO functional enrichment bubble plot of upregulated differentially expressed genes; (**b**) GO functional enrichment bubble plot of downregulated differentially expressed genes; (**c**) KEGG functional enrichment classification annotation chart of upregulated differentially expressed genes; (**d**) KEGG functional enrichment classification annotation chart of downregulated differentially expressed genes.


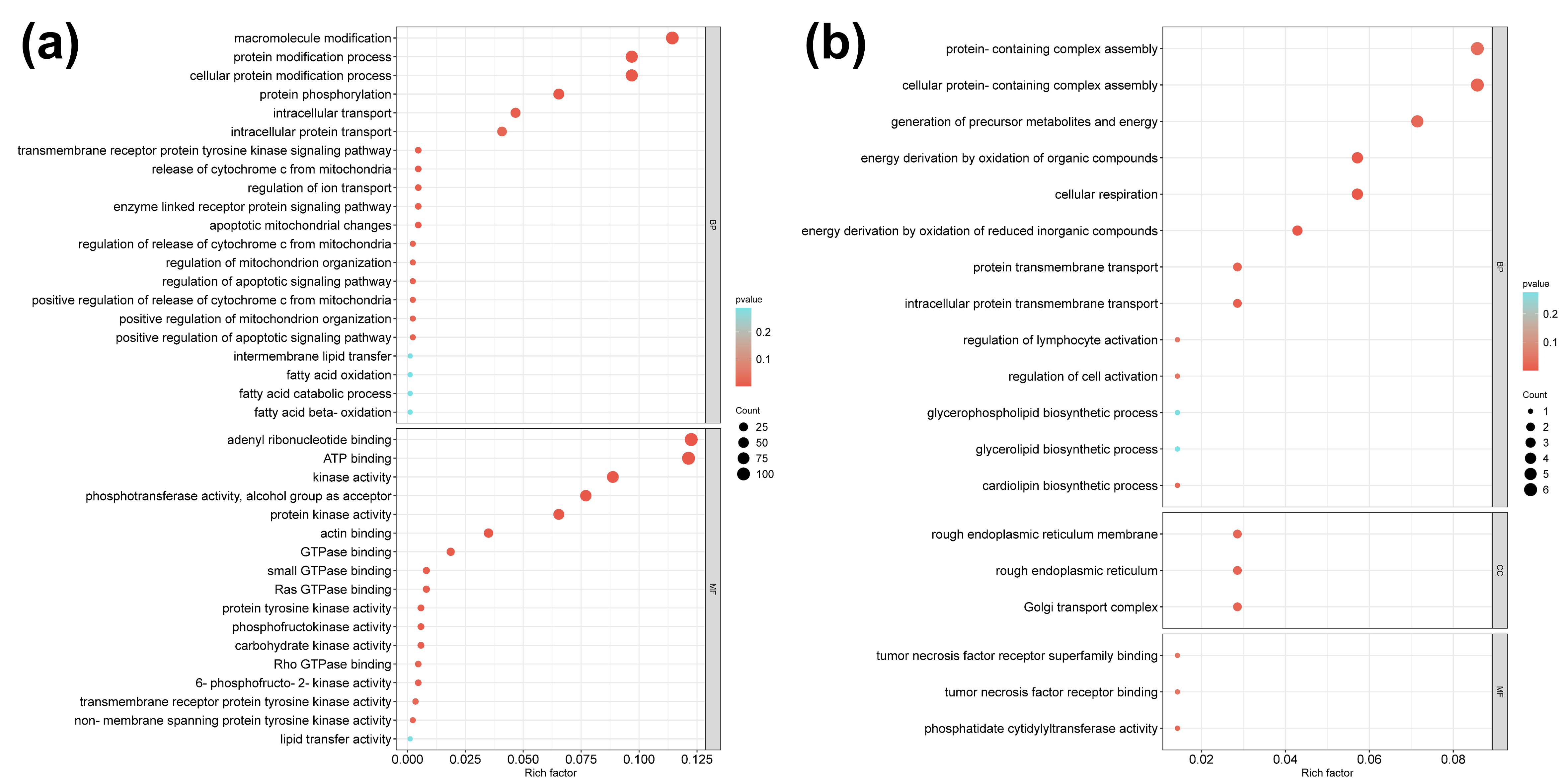


**Supplementary Figure S6.** Functional enrichment analysis of CO_IMAdCs2 vs CO_SMSCs2 group. (**a**) GO functional enrichment bubble plot of upregulated differentially expressed genes; (**b**) GO functional enrichment bubble plot of downregulated differentially expressed genes.


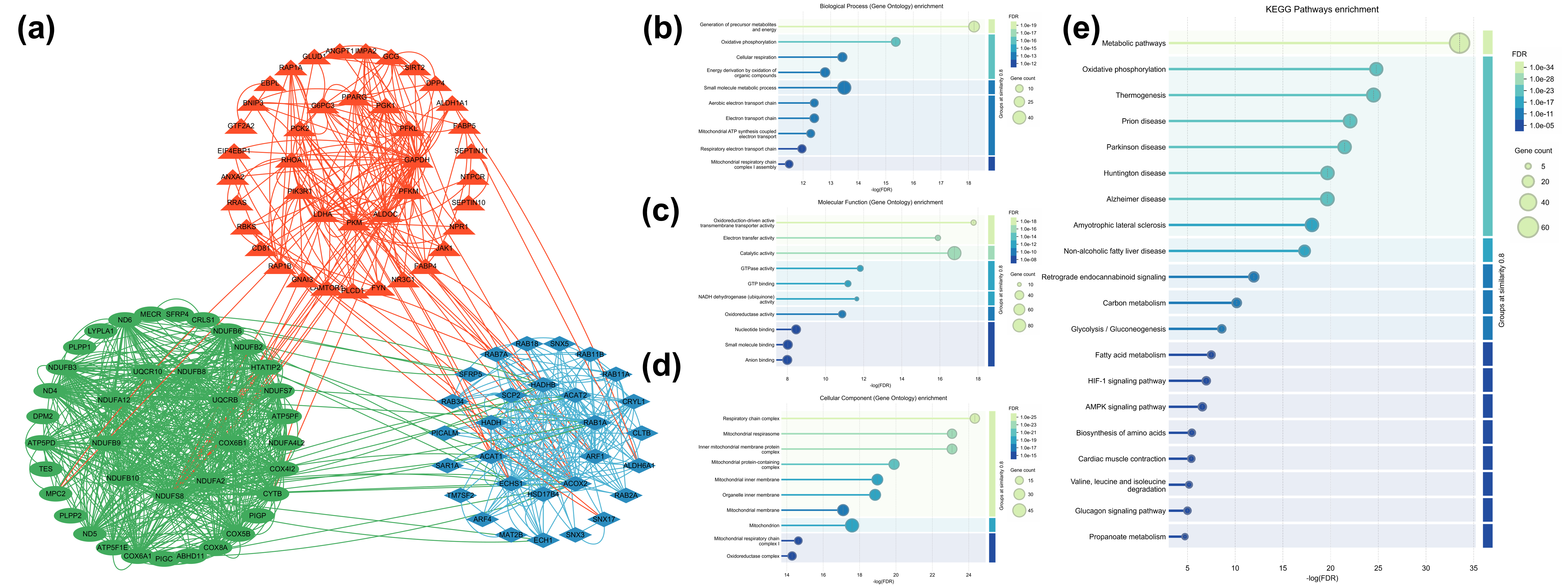


**Supplementary Figure S7.** Analysis of protein interaction networks and functional enrichment of key differential genes up-regulated in the IMAdCs2 vs CO_ IMAdCs2 group. (**a**) PPI network of up-regulated key differential genes in the IMAdCs2 vs CO_ IMAdCs2 group, divided into three major sub-network modules of red, green and blue using k-means clustering method; (**b-d)**: Functional enrichment analysis in IMAdCs2 vs CO_ IMAdCs2 group up-regulated key differential genes related to biological processes, molecular functions and cellular components, highlighting metabolic pathways such as energy production and oxidative phosphorylation; (**e**) KEGG pathway enrichment analysis of up-regulated key differential genes in the IMAdCs2 vs CO_ IMAdCs2 group, highlighting notable functional pathways such as metabolic pathways, oxidative phosphorylation, thermogenesis, NAFLD and AMPK signalling pathways.


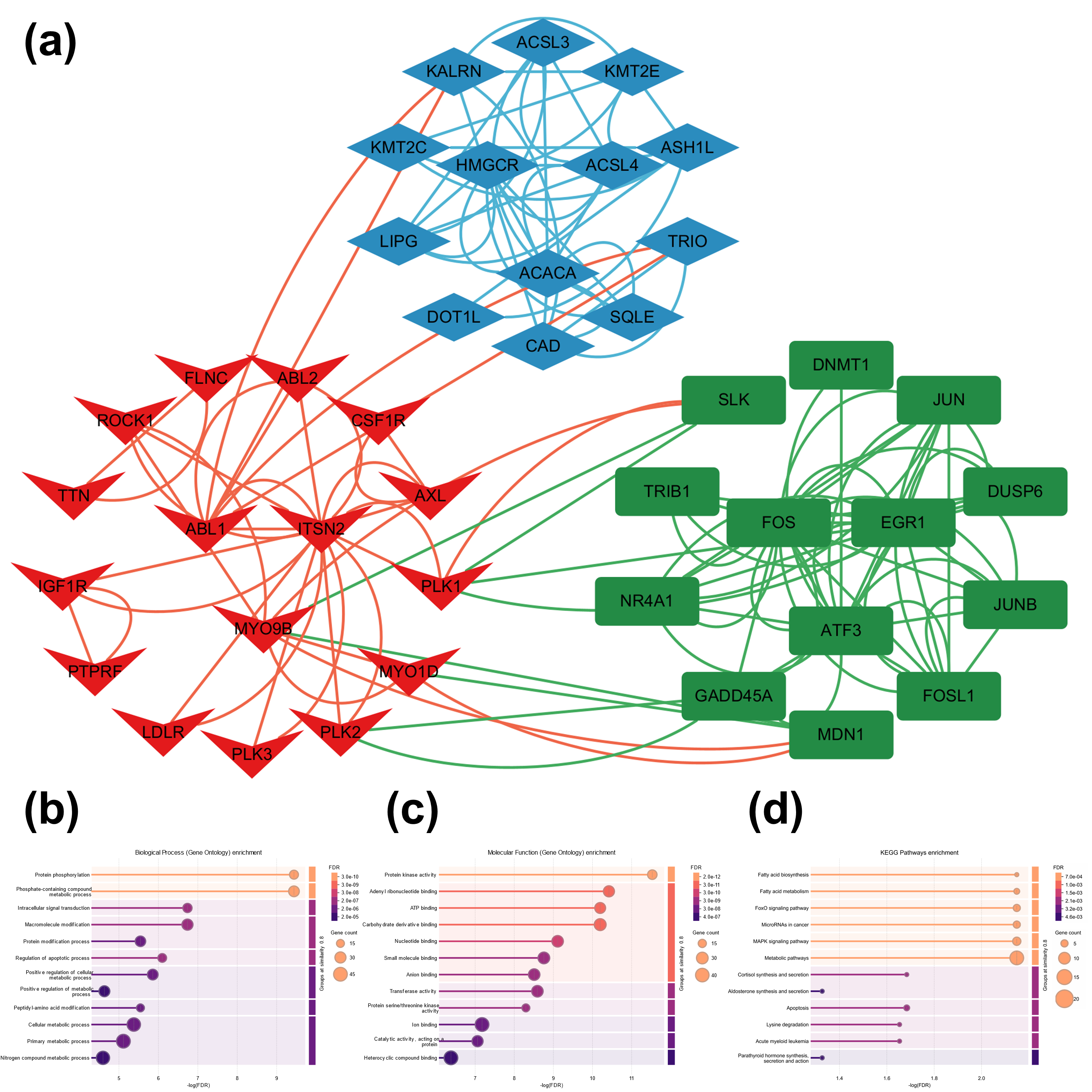


**Supplementary Figure S8.** Analysis of protein interaction networks and functional enrichment of key differential genes down-regulated in the IMAdCs2 vs CO_ IMAdCs2 group. (**a**) PPI network of down-regulated key differential genes in the IMAdCs2 vs CO_ IMAdCs2 group, divided into three major sub-network modules of red, green and blue using k-means clustering method; (**b, c**): Functional enrichment analysis of the IMAdCs2 vs CO_ IMAdCs2 group of down-regulated key differential genes associated with biological processes and molecular functions; (**d**) KEGG pathway enrichment analysis of down-regulated key differential genes in the IMAdCs2 vs CO_ IMAdCs2 group.


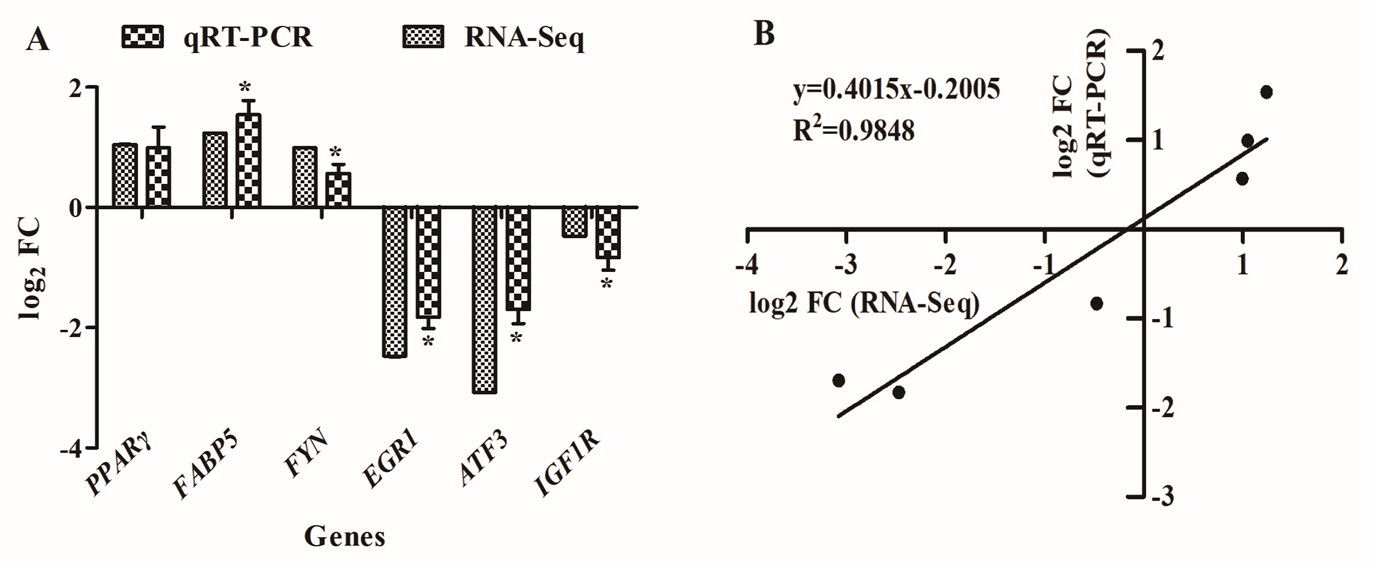


**Supplementary Figure S9. qRT-PCR verifies screening for differentially expressed genes. (A)** The differentially expressed genes were confirmed by qRT-PCR; (**B)** The correlation of fold change of differential expression between the RNA-seq and qRT-PCR. A paired two-tailed Student’s t-test was used. * *P* < 0.05.
